# The Ethical Aspects of Medical Consultation

**Published:** 1907-05-25

**Authors:** 


### The Hospital

A Journal of

The Medical Sciences and Hospital Administration.

New Series. No. 8, Vol. I. [No. 1030, Vol. XLII.] SATURDAY, MAY 25, 1907.

### THE ETHICAL ASPECTS OF MEDICAL CONSULTATION.

The various "Divisions" of the British Medical Association have recently been invited by the Ethical Committee of the Association to consider a special report on the subject of consultants and professional consultations, and to vote "aye" or "no" on a series of specific recommendations attached to the report. In due course the decisions, whatever they may be, will come before the representative meeting, and it is anticipated that in this way will be formulated a code of rules which will exercise control, on the subjects with which they deal, over the practice and custom of the members of the Association. It is premature to speculate on the issue of the discussion, but it is manifest that it may have important consequences both for the profession and the public, and thus it is well that the recommendations of the Ethical Committee should be most carefully examined. There can be no question that in connection with professional consultations positions of some delicacy, involving both ethical and personal considerations, do sometimes arise, and hence it may be allowed that an attempt to frame rules to meet the various possibilities of difficulty and controversy is a legitimate and proper proceeding. All that need be added to this general remark is that such rules must not be unduly rigid, and obviously they must in no wise obscure the very first claim of medical practice, the safety and welfare of the patient. The public mind is for the most part in a very much befogged condition in regard to a supposed code of medical etiquette, which, by the way, is mainly the creation of its own combined imagination and ignorance, and it is not in the interest of the profession to increase this mental confusion. But there can be no objection to the framing of rules which will increase smooth and dignified working between members of the profession themselves, and will, at the same time, recognise that the profession exists for the purpose of public ministry and service.

The first suggestion in the Ethical Committee's report which calls for discussion is the proposal to establish the formal recognition of a distinct class of practitioners to be specifically designated, "Consultants." The qualification for this class appears to be the restriction of practice to patients introduced by other practitioners, and abstinence from regular attendance on any cases. There is, however, no indication of the provision of any machinery by which these proposals can acquire actuality. What body or authority, for example, will have power to permit or authorise the use of the designation, and how are claims to it to be examined or established? Suppose, again, that a practitioner adopts and professes the term "Consultant," and it is subsequently urged that he has, in one or more instances, acted inconsistently with the terms of its definition. Is he to be liable to pains and penalties? And, if so, how are the necessary inquiries to be conducted and the punishment, if any, enforced? These and related questions will inevitably arise if the proposal is adopted, and if, which is more than doubtful, anyone troubles to take the slightest notice of it. But even were it possible to make the proposal effective, we question whether it would be advisable to do so. There is about it a rigidity and inelasticity which hardly accommodate themselves to the duties and responsibilities and varying conditions of professional life. The broad position, as generally, and we believe wisely, recognised at the present day, is that every qualified medical practitioner is free to choose whatever line of professional work he desires. He is "legally qualified" in all departments of practice, and no one has the right to restrict his activities. Of course this implies that it is his duty to equip himself to the level of his pretensions, and he must needs accept responsibility both for his professions and his activities. But there is no right or title to forbid his cultivation of any opportunities which come to his hand, and no one may confine him within the borders of any special or restricted province. With all this it is common knowledge that certain practitioners have acquired special experience in particular departments, and to these, naturally, cases of doubt or difficulty in such departments become referred by their *confrères*. All this is going on with little or no trouble, and the general practitioner is perfectly well able to distinguish the real from the pretended consultant. To propose the institution of a special designation to aid in this distinction is more likely to cause confusion than to afford help, for it is not improbable that the term will be mainly flaunted by those who are least qualified to bear it. The fact is that no amount of affirmation or restriction can make a practitioner in any real sense a consultant. Such a position depends on the recognition of special knowledge or ability by those competent to judge; that is, by other members of the profession. Hence the proposal that a practitioner who professes a certain rule of practice, and who apparently may be innocent of all experience, shall have the right to adopt the term "consultant," is little short of ludicrous, and is calculated to mislead the public, and even in some instances members of the profession. Further, such a proposal involves a slight on many senior practitioners who, engaged for years in general practice, come to be recognised by their colleagues and neighbours as capable of affording valuable help and counsel in many circumstances of difficulty. Men of this type and quality enjoy a high measure of respect and regard, and they represent to the public a welcome and most effective presentation of the capacity of the profession. Is it to be said that because these gentlemen have their own private patients they cannot claim a rank and distinction which may, perchance, be the boast of the junior member of the local hospital staff? The question is to be answered by the logic of facts, and not by the provision of artificial definitions. Medicine has already a sufficient confusion of names and titles, and we cannot think the profession will welcome a suggestion to increase the number.

Short of the preliminary proposition above discussed, we have no very urgent objection to the Ethical Committee's proposals. Some of them will not easily be enforced, even though the principle they contain is sound, and many of them suggest what hardly needs suggestion where courtesy and a sense of responsibility exist. But if there are men who will be helped by the announcement that punctuality is a virtue, and that it is polite to acknowledge the receipt of a courteously expressed letter, by all means let us have these and similar inspiring precepts duly supported by the fiat of the British Medical Association.

